# Multi-modality biomarkers in the early prediction of ischaemic heart disease in middle-aged men during a 21-year follow-up

**DOI:** 10.1186/s12872-021-01886-x

**Published:** 2021-02-02

**Authors:** Maria Sakalaki, Per-Olof Hansson, Annika Rosengren, Erik Thunström, Aldina Pivodic, Michael Fu

**Affiliations:** 1 Department of Molecular and Clinical Medicine, Institute of Medicine, Sahlgrenska Academy, Sahlgrenska University Hospital/Östra Hospital, University of Gothenburg, Diagnosvägen 11, 41650 Gothenburg, Sweden; 2 Department of Medicine, Geriatrics and Emergency Medicine, Sahlgrenska University Hospital/Östra, Gothenburg, Sweden; 3 Statistiska Konsultgruppen, Gothenburg, Sweden; 4 Department of Ophthalmology, Institute of Neuroscience and Physiology, Sahlgrenska Academy, University of Gothenburg, Gothenburg, Sweden

**Keywords:** Prevention, Ischaemic heart disease, Risk factors

## Abstract

**Background:**

Ischaemic heart disease (IHD) often develops after decades of preceding subclinical coronary atherosclerosis. Biomarkers are useful prognostic predictors of IHD, but their long-term predictive value in a general population has not been adequately studied.

**Purpose:**

To investigate the early predictive value of multi-modality biomarkers in addition to clinical risk factors in incident IHD in a random male general population sample followed from 50 to 71 years of age.

**Method:**

*“The Study of Men Born in 1943”* is a longitudinal cohort study during follow-up. All the men underwent a baseline examination in 1993, where a panel of biomarkers were analysed and incident IHD was registered during 21-year follow-ups.

**Results:**

Of 739 participants, 97 men (13.1%) developed an IHD event. For time to first occurrence of IHD, univariable analyses showed that elevated levels of high sensitivity troponin T (hs-TNT), high sensitivity-C reactive protein (hs-CRP) and interleukin-6 (IL-6) were significant predictors of IHD. In addition, a high number of biomarkers with elevated levels (hs-TNT > 10 ng/L, hs-CRP > 1 mg/L, IL-6 > 8 ng/L and N-terminal pro b-type natriuretic peptide (NT-proBNP) > 100 pg/mL) increased predictive ability. In univariable and multivariable analysis high-density lipoprotein-cholesterol (HDL-C) had the highest predictive ability. Hs-TNT provided better predictive ability than smoking, body mass index and glucose, and was an independent significant predictor when adjusted for HDL-C, total cholesterol and hypertension. Addition of biomarkers on top of clinical risk factors provided significantly better prediction as tested by likelihood ratio test (p = 0.033), but did not significantly enhance the model’s discriminative ability However, it appeared contributing to higher sensitivity in the late phase of follow-up.

**Conclusion:**

In this random, middle-aged male population sample, the addition of biomarker hs-TNT was an independent significant predictor of IHD and significantly improved prediction, indicating the probability of a better prediction of long-term risk of IHD in a low-risk population.

*Trial registration*: The study is registered at Clinical Trials.gov Identifier number: NCT03138122

## Introduction

Atherosclerosis develops gradually over a period of decades and its progress depends on several components such as hypercholesterolemia, lipid oxidation and inflammation [1]. Several inflammatory components take part in the pathogenesis of atherosclerosis [2, 3] and, of them, interleukin-6 (IL 6) and high sensitivity-C reactive protein (hs-CRP) are the most extensively studied. To date hs-CRP measurements are recommended for the assessment of cardiovascular risk in some asymptomatic adults, in spite of the fact that causality in ischaemic heart disease (IHD) has not been established [4].

Natriuretic peptides are released from the heart as a reaction to increased wall stress, pressure and hypertrophy and have been studied as prognostic biomarkers in IHD [5–7]. Jørgesen et al. have previously shown that N-terminal pro b-type natriuretic peptide (NT- proBNP) in combination with changes in electrocardiography predicted all-cause mortality and cardiovascular events in patients without previous cardiac disease [8], but the predictive value of natriuretic peptides on the incidence of IHD has not been adequately studied.

Little is known about the significance and consequences of elevated troponin in the general population, despite the fact that troponin is commonly used to establish diagnosis and prognosis in myocardial infarction. Daniels et al. showed that a population of community-dwelling older adults (mean age 77 years) with elevated troponin T (TNT) (≥ 0.01 ng/ml) and NT-proBNP (≥ 450 pg/ml) ran a higher risk of both all-cause mortality and cardiovascular mortality during a median follow-up of 6.8 years [9]. A combination of biomarkers and traditional risk factors has previously been studied in terms of the prediction of death and major adverse cardiovascular events, showing different predictive abilities for increased risk [10–12].

The objective of the present study was to investigate the additive value of a panel of biomarkers on top of clinical risk factors in the early prediction of the incidence of an IHD in a cohort of randomly selected male sample from the general population, investigated at 50 years of age and followed for 21 years. We hypothesised that while, clinical risk factors may be of limited predictive value for incident IHD in the general population, the addition of multiple biomarkers will resemble the complex and multifactorial nature of the atherosclerotic process and therefore facilitate the early identification of high-risk individuals in a general population.

## Methods

### Study design

The “*Study of Men Born In 1943”* is a longitudinal cohort study initiated in 1993, investigating cardiovascular risk factors and diseases. The cohort sample was randomly selected, comprising half of all men born in 1943 and living in the City of Gothenburg, Sweden. An invitation to the study was sent to 1463 persons, of which 798 (55%) accepted participation in the study and underwent a health examination at entry.

The health examination included a physical examination, blood testing and questionnaires on medical history, lifestyle, physical activity and mental well-being. All the examinations were conducted at Sahlgrenska University Hospital, Gothenburg, Sweden.

### Examinations

Leisure time physical activity was assessed using the Saltin-Grimby questionnaire [13] and coded as 1 = sedentary (physically inactive); 2 = some light physical activity such as walking, riding a bicycle and light gardening for at least four hours a week; 3 = regular moderate physical activity for a minimum of three hours a week and 4 = regular hard physical training for competition sports. Men who were current smokers or had quit smoking less than one month before the examination were categorised as smokers. A former smoker was defined as having ceased smoking more than one month ago, while a never smoker was defined as someone that had never used cigarettes or cigars or smoked a pipe regularly; in the risk factor analyses, these two categories were grouped together as they showed a similar impact on the studied endpoint.

Body weight was measured to the nearest 0.1 kg and was recorded with the participant wearing light indoor clothing. Height was recorded to the nearest centimetre while the participant was standing barefoot. Body mass index (BMI) was calculated as weight (kg)/height (m)^2^. Blood pressure was recorded in the right arm in a sitting position using a standard mercury sphygmomanometer. Hypertension was defined as either a medical history with current anti-hypertensive medication or present blood pressure, systolic (SBP) ≥ 140 and/or diastolic (DBP) ≥ 90 mmHg. Diabetes was defined as fasting blood-glucose of > 7 mmol/L, or using oral medication or insulin, or reported diabetes at the initial examination. Hyperlipidaemia was defined as total cholesterol of > 6.2 mmol/L or using lipid-lowering medication.

Fasting venous blood samples were drawn before each clinical examination and plasma levels of glucose, total serum cholesterol and high-density lipoprotein cholesterol (HDL-C) were analysed using standard laboratory procedures. A panel of biomarkers was analysed (Elecsys, Roche) from blood samples drawn at the visit in 1993 and kept frozen (minus 70 °C) until analysis in 2014. The analysis included high-sensitivity troponin T (hs-TNT), IL-6, hs-CRP and NT-proBNP. In addition to the analyses performed on the continuous scale of biomarkers, dichotomous ones were examined; they included hs-TNT > 3 ng/L, hs-TNT > 5 ng/L, hs-TNT > 10 ng/L, IL-6 > 8 ng/L, hs-CRP > 1 mg/L, hs-CRP > 2 mg/L, hs-CRP > 3 mg/L, NT-proBNP > 50 pg/mL and NT-proBNP > 100 pg/mL. The dichotomous values were chosen to be within different levels of the normal reference range, because the cohort sample was randomly selected from the general population and assumed to be generally healthy. The definition of the number of modality biomarkers included hs-CRP > 1 mg/L, NT-proBNP > 100 pg/mL, hs-TNT > 10 ng/L and IL-6 > 8 ng/L and ranged between 0 and 4.

### Definition of IHD

A data file relating to the participants was matched against the Swedish National Patient Register and the Swedish National Death Register identifying International Statistical Classification of Disease and Related Health Problems (ICD) codes I20-21 (ICD 10) and 410–414 (ICD 9). In order to confirm and validate the diagnoses, medical records from hospitals in Gothenburg were collected and scrutinised. IHD was defined as (1) new onset of myocardial infarction fatal or non-fatal, (2) hospitalised unstable angina or (3) intervention with either percutaneous coronary intervention (PCI) or coronary artery bypass graft (CABG) from the participants’ first examination in 1993 to 30 August 2014, whichever occurred first. Prior IHD was defined as an IHD event prior to the first examination for each participant.

### Statistical methods

All the analyses were performed using SAS software version 9.4 (SAS Institute Inc., Cary, NC, USA). Baseline characteristics were presented as number and percentage and mean ± standard deviation (± SD), or median and range (minimum–maximum), where appropriate. For comparisons between groups of incident and non-incident IHD, Fisher’s exact test was used for dichotomous variables, the Mantel–Haenszel chi-square test for ordered categorical variables and the Mann–Whitney U-test for continuous variables. Event rates per 1000 person-years were calculated as the number of events divided by the number of follow-up years multiplied by 1000. Ninety-five (95) % confidence intervals (CI) were estimated using exact Poisson limits. The impact of clinical risk factors and biomarkers on the first incidence of IHD were studied applying univariable Cox proportional hazards models.

Framingham 10-year risk score on the basis of age, total cholesterol, HDL cholesterol, SBP, treatment for hypertension and smoking status was calculated in the current cohort for comparison [14].

The effect size was presented as the hazard ratio (HR) with 95% CI. The association of the variables impact was ranked through standardized HR that is HR expressed by 1 SD increase or decrease (gradient of risk). Model A included only the calculated Framingham 10-year risk score. Model B was developed keeping significant variables from the stepwise regression, forward and backward choosing the model with the lowest Akaike Information Criterin (AIC) when the two methods selected two different models. The variables included were the univariably significant known clinical risk factors for IHD, total cholesterol, HDL cholesterol, hyperlipidemia, SBP, DBP, hypertension and smoking. In the model C, significant factors from the univariable analyses of glucose, BMI, physical activity and biomarkers were added. Improvement of model C vs model B was tested by performing likelihood ratio test. The probability of experiencing an IHD from the age of 50 during 21 years of follow-up was calculated using each model and their discriminative ability was described by the area under received operating characteristic curve (ROC). An area under the ROC curve (AUC) of > 0.70 is considered acceptable. The ROC curves were compared applying the non-parametric approach of DeLong, DeLong and Clarke-Pearson implemented in the SAS logistic procedure. Additionally, time-dependent AUCs were obtained along the follow-up time using SAS phreg procedure. Internal validation was performed applying tenfold cross-validation for the selected model. The model’s ability of correctly estimating the IHD probabilities was evaluated by calibration plots. The probability cut-offs optimizing sensitivity and specificity were identified using Youden’s index (sensitivity + specificity-1).

The proportional hazards assumptions were checked by evaluating the interactions between logarithmic time in study and the studied variables in the Cox regression analyses. In the univariable analyses, interaction with time was observed for some variables (increase in Framingham risk score, all variables for hs-CRP, IL-6 > 8, number of risk biomarkers and decrease in HDL) with a negative association, suggesting that the relative estimates declined during the follow-up period. However, in the risk model C, this assumption was fulfilled. All tests were two-tailed and a *p*-value of < 0.05 was regarded as statistically significant. One HDL cholesterol value of 0.06, considered as not valid value, was set to missing in all analyses. Missing data were not imputed.

## Results

Of 798 participants, 59 (7.5%) were excluded in total, of which 45 (5.6%) were excluded due to the lack of biomarker data and 14 (1.8%) due to the occurrence of an IHD event prior to baseline examination. A total of 739 (92.6%) participants of the original sample were therefore included in the current study.

During a median follow-up of 20.6 (range 0.6–21.6) years, 97 participants (13.1%) had an incident event of myocardial infarction (MI), unstable angina, PCI or CABG. The IHD events were divided as follows: MI with or without intervention with PCI or CABG (62.9%), PCI or CABG due to angina (27.8%) and hospitalised unstable angina (9.3%). The event rate was 7.0 [95% CI 5.8–8.6] per 1000 person-years. A total of 105 (14.2%) participants died, including all causes, before the end of follow-up on 30 Aug 2014. Of these, 33 (31.4%) died of cardiovascular causes and 72 (68.6%) died of other causes.

### Baseline characteristics

As shown in Table 1, 30.9% of the participants were smokers, 39.4% had hypertension and 35.6% had hyperlipidaemia. Hypertension was more common among those that developed IHD (57.7% vs 36.6%, *p*-value 0.0001). Total cholesterol (6.19 vs 5.83, *p*-value 0.0043) and BMI (27.0 vs 26.1 kg/m^2^, *p*-value 0.022) were higher in the group with IHD events. HDL cholesterol was lower in the group with IHD compared to non-IHD (1.16 vs 1.33, *p*-value < 0.0001). No significant difference was seen in the prevalence of diabetes, hyperlipidaemia, smoking and physical activity.

**Table 1:** Patients characteristics from 1993 by incidence of MI/Unstable angina/PCI/CABG during 21-years of follow-up

	Total (n = 739)	No MI, unstable angina, PCI or CABG (before 20,140,830) (n = 642)	MI, unstable angina, PCI or CABG (before 20,140,830) (n = 97)	*p* value
Framingham score	11.0 (2.2) 11.0 (5.0; 18.0) n = 707	10.9 (2.1) 11.0 (5.0; 17.0) n = 616	12.3 (2.1) 12.0 (7.0; 18.0) n = 91	< .0001
Total cholesterol (mmol/L)	5.88 (1.04) 5.80 (3.10; 9.90) n = 739	5.83 (1.03) 5.80 (3.10; 9.10) n = 642	6.19 (0.99) 6.00 (4.20; 9.90) n = 97	0.0043
Hyperlipidemia	263 (35.6%)	222 (34.6%)	41 (42.3%)	0.18
HDL cholesterol	1.31 (0.34) 1.30 (0.57; 3.70) n = 710	1.33 (0.34) 1.30 (0.57; 3.70) n = 619	1.16 (0.31) 1.10 (0.58; 2.40) n = 91	< .0001
Systolic blood pressure (mmHg)	128.9 (17.3) 128.0 (92.0; 198.0) n = 736	128.0 (17.0) 126.0 (92.0; 198.0) n = 639	134.4 (17.9) 135.0 (92.0; 178.0) n = 97	0.0003
Diastolic blood pressure (mmHg)	84.4 (10.6) 84.0 (56.0; 120.0) n = 736	83.9 (10.5) 82.0 (56.0; 120.0) n = 639	87.3 (11.0) 88.0 (62.0; 110.0) n = 97	0.0027
Hypertension	291 (39.4%)	235 (36.6%)	56 (57.7%)	0.0001
Blood pressure lowering medication	44 (6.0%)	29 (4.5%)	15 (15.5%)	0.0004
Smoker	228 (30.9%)	191 (29.8%)	37 (38.1%)	0.12
BMI (kg/m^2^)	26.2 (3.4) 25.8 (16.6; 41.6) n = 739	26.1 (3.4) 25.7 (16.6; 41.6) n = 642	27.0 (3.6) 26.7 (18.5; 38.3) n = 97	0.022
Fasting plasma glucose (mmol/L)	4.67 (1.26) 4.50 (2.60; 19.20) n = 736	4.63 (1.16) 4.50 (2.60; 19.20) n = 639	4.95 (1.80) 4.60 (3.80; 17.60) n = 97	0.0059
Diabetes	25 (3.4%)	20 (3.1%)	5 (5.2%)	0.44
Sedentary leisure time	112 (15.2%)	92 (14.3%)	20 (20.6%)	0.15
High-sensitivity C-reactive Protein (mg/L)	2.26 (3.60) 1.23 (0.14; 47.06) n = 739	2.22 (3.47) 1.21 (0.14; 47.06) n = 642	2.51 (4.40) 1.41 (0.16; 40.25) n = 97	0.12
hsCRP > 1 mg/L	448 (60.6%)	378 (58.9%)	70 (72.2%)	0.015
hsCRP > 2 mg/L	240 (32.5%)	207 (32.2%)	33 (34.0%)	0.81
hsCRP > 3 mg/L	135 (18.3%)	118 (18.4%)	17 (17.5%)	0.97
NT-proBNP (pg/mL)	33.9 (40.7) 23.7 (5.0; 375.2) n = 739	33.8 (40.1) 24.0 (5.0; 375.2) n = 642	34.3 (44.8) 22.1 (5.0; 291.4) n = 97	0.98
NT-proBNP > 50 pg/mL	132 (17.9%)	117 (18.2%)	15 (15.5%)	0.62
NT-proBNP > 100 pg/mL	41 (5.5%)	36 (5.6%)	5 (5.2%)	1.00
High-sensitivity Troponin-T (ng/L)	5.07 (3.30) 4.04 (3.00; 38.96) n = 739	4.97 (3.10) 3.98 (3.00; 37.20) n = 642	5.67 (4.35) 4.49 (3.00; 38.96) n = 97	0.041
hsTroponin-T > 3 ng/L	507 (68.6%)	431 (67.1%)	76 (78.4%)	0.032
hsTroponin-T > 5 ng/L	265 (35.9%)	225 (35.0%)	40 (41.2%)	0.28
hsTroponin-T > 10 ng/L	39 (5.3%)	29 (4.5%)	10 (10.3%)	0.045
IL-6 (ng/L)	2.77 (3.90) 1.88 (1.50; 87.10) n = 736	2.70 (3.84) 1.84 (1.50; 87.10) n = 641	3.21 (4.26) 2.25 (1.50; 36.61) n = 95	0.023
IL-6 > 8 ng/L	19 (2.6%)	16 (2.5%)	3 (3.2%)	0.91
Number of risk biomarkers (hsCRP > 1,NT-proBNP > 100,hsTroponin-T > 10,IL6 > 8)				
0	264 (35.7%)	240 (37.4%)	24 (24.7%)	
1	408 (55.2%)	349 (54.4%)	59 (60.8%)	
2	62 (8.4%)	49 (7.6%)	13 (13.4%)	
3	5 (0.7%)	4 (0.6%)	1 (1.0%)	0.0053

For categorical variables n (%) is presented

For continuous variables Mean (SD) / Median (Min; Max) / n = is presented

For comparison between groups Fisher´s Exact test (lowest 1-sided p-value multiplied by 2) was used for dichotomous variables and the Mantel–Haenszel Chi Square test was used for ordered categorical variables and the Mann–Whitney U-test was used for continuous variables

The serum levels of the biomarkers are shown in Table 1. The median concentration of hs-CRP mg/L was 1.23 (range 0.14–47.06), hs-TNT median 4.04 ng/L (range 3.00–38.96), NT-pro-BNP pg/mL median 23.7 (range 5.0–375.2), IL-6 median 1.88 ng/L (range 1.50–87.10) and fasting blood glucose mean 4.67 mmol/L (± 1.26). Biomarkers hs-TnT, IL-6, hs-CRP > 1 mg/L, number of risk biomarkers and glucose were significantly higher in the group with an IHD event in comparison with those without, Table 1.

### Univariable analyses

As shown in Table 2, by using univariable Cox proportional hazards models, several known risk factors and investigated biomarkers were associated with an increased risk of incidence of an IHD event. As a single risk factor, HDL cholesterol had the highest predictive value according to the ordering of gradient of risk. A decrease in HDL cholesterol of 0.2 units (mmol/L) increased the risk of an IHD event by 45% (HR 1.45 (95% CI 1.24–1.69, p < 0.0001). The second highest predictive value was found for hypertension, HR 2.30 (95% CI 1.53–3.44, *p* < 0.0001). The number of risk biomarkers with elevated levels, HR 1.64 (95% CI 1.21–2.21, *p* = 0.0014) per one biomarker with elevated level, was the strongest predictor among studied biomarker variables. Other significant predictors in the univariable analysis were: SBP, total cholesterol, DBP, hs-CRP > 1 vs ≤ 1 mg/L, categorized hs-TNT, hs-TNT > 3 vs ≤ 3 ng/L, BMI, glucose, smoking, hs-TNT > 10 vs ≤ 10 ng/L, hs-TNT (as continuous variable) and IL-6 (as continuous variable). Among the significant predictors, increase in the combined biomarker variable (*p* = 0.0016), increase in hs-CRP > 1 mg/L (*p* = 0.0069), and decrease in HDL cholesterol (*p* = 0.0013) showed a negative interaction with follow-up time in the study, indicating greater impact on the incidence of IHD at the beginning of the study follow-up and a decline over time.

**Table 2:** Cox proportional hazards model for time to first of MI/Unstable angina/PCI/CABG during 21 years of follow-up

				Univariable model			Gradient of risk (standardized Hazard ratio by 1 SD)		
Predictors		n (%) events	N	Hazard ratio (95% CI)	*p*-value	PH *p* value	SD	Hazard ratio (95% CI)	Effect ordering
Total cholesterol (mmol/L)	Risk by 1 unit increase	97 (13.1%)	739	1.34 (1.11–1.61)	0.0022	0.70	1.04	1.35 (1.12–1.64)	5
Hyperlipidemia	No hyperlipidemia	56 (11.8%)	739						
	Hyperlipidemia	41 (15.6%)		1.34 (0.89–2.00)	0.16	0.44	0.48	1.15 (0.95–1.39)	
HDL cholesterol (mmol/L)	Risk by 0.2 units decrease	91 (12.8%)	710	1.45 (1.24–1.69)	< .0001	0.0013 Neg	0.34	1.89 (1.45–2.45)	1
Systolic BP (mmHg)	Risk by 1 unit increase	97 (13.2%)	736	1.02 (1.01–1.03)	0.0006	0.78	17.25	1.36 (1.14–1.63)	4
Diastolic BP (mmHg)	Risk by 1 unit increase	97 (13.2%)	736	1.03 (1.01–1.05)	0.0024	0.51	10.61	1.34 (1.11–1.63)	6
Hypertension	No hypertension	41 (9.2%)	739						
	Hypertension	56 (19.2%)		2.30 (1.53–3.44)	< .0001	0.54	0.49	1.50 (1.23–1.83)	2
Smoking	Non-smoker	60 (11.7%)	739						
	Smoker	37 (16.2%)		1.54 (1.02–2.32)	0.040	0.062	0.46	1.22 (1.01–1.47)	13
BMI (kg/m2)	Risk by 1 unit increase	97 (13.1%)	739	1.07 (1.01–1.13)	0.018	0.21	3.42	1.25 (1.04–1.50)	11
Fasting plasma glucose (mmol/L)	Risk by 1 unit increase	97 (13.2%)	736	1.17 (1.05–1.30)	0.0040	0.43	1.26	1.22 (1.06–1.39)	12
Physical activity	Moderate or regular exercise	77 (12.3%)	739						
	Sedentary leisure time	20 (17.9%)		1.50 (0.92–2.45)	0.11	0.55	0.36	1.16 (0.97–1.38)	
High-sensitivity C-reactive Protein (mg/L)	Risk by 5 unit increase	97 (13.1%)	739	1.13 (0.91–1.40)	0.25	0.0025 Neg	3.60	1.09 (0.94–1.28)	
hsCRP > 1 mg/L	≤ 1 mg/L	27 (9.3%)	739						
	> 1 mg/L	70 (15.6%)		1.82 (1.17–2.84)	0.0080	0.0069 Neg	0.49	1.34 (1.08–1.67)	7
hsCRP > 2 mg/L	≤ 2 mg/L	64 (12.8%)	739						
	> 2 mg/L	33 (13.8%)		1.15 (0.76–1.75)	0.52	0.0007 Neg	0.47	1.07 (0.88–1.30)	
hsCRP > 3 mg/L	≤ 3 mg/L	80 (13.2%)	739						
	> 3 mg/L	17 (12.6%)		1.02 (0.61–1.73)	0.93	0.0031 Neg	0.39	1.01 (0.82–1.23)	
NT-proBNP (pg/mL)	Risk by 20 unit increase	97 (13.1%)	739	1.02 (0.92–1.12)	0.73	0.20	40.72	1.04 (0.85–1.26)	
NT-proBNP > 50 pg/mL	≤ 50 pg/mL	82 (13.5%)	739						
	> 50 pg/mL	15 (11.4%)		1.13 (0.65–1.96)	0.66	0.77	0.38	1.05 (0.85–1.30)	
NT-proBNP > 100 pg/mL	≤ 100 pg/mL	92 (13.2%)	739						
	> 100 pg/mL	5 (12.2%)		1.00 (0.41–2.47)	0.99	0.39	0.23	1.00 (0.81–1.23)	
High-sensitivity Troponin-T (ng/L)	Risk by 1 unit increase	97 (13.1%)	739	1.05 (1.01–1.10)	0.028	0.23	3.30	1.17 (1.02–1.35)	15
High-sensitivity Troponin-T > 3 (ng/L)	≤ 3	21 (9.1%)	739						
	> 3	76 (15.0%)		1.76 (1.09–2.86)	0.021	0.85	0.46	1.30 (1.04–1.63)	9
High-sensitivity Troponin-T > 5 (ng/L)	≤ 5	57 (12.0%)	739						
	> 5	40 (15.1%)		1.31 (0.87–1.96)	0.19	0.34	0.48	1.14 (0.94–1.38)	
High-sensitivity Troponin-T > 10 (ng/L)	≤ 10	87 (12.4%)	739						
	> 10	10 (25.6%)		2.33 (1.21–4.48)	0.011	0.39	0.22	1.21 (1.04–1.40)	14
High-sensitivity Troponin-T ≤ 3/3–10/ > 10 (ng/L)	≤ 3	21 (9.1%)	739						
	> 3–10	66 (14.1%)		1.65 (1.01–2.69)	0.047	0.71	0.48	1.27 (1.00–1.61)	10
	> 10	10 (25.6%)		3.31 (1.56–7.04)	0.0018	0.59	0.22	1.30 (1.10–1.54)	8
IL-6	Risk by 5 unit increase	95 (12.9%)	736	1.17 (1.00–1.36)	0.044	0.71	3.90	1.13 (1.00–1.27)	16
IL-6 > 8	≤ 8	92 (12.8%)	736						
	> 8	3 (15.8%)		1.46 (0.46–4.61)	0.52	0.034 Neg	0.16	1.06 (0.88–1.27)	
Number of risk biomarkers with elevated levels (hsCRP > 1/NT-proBNP > 100/hsTroponin-T > 10/IL6 > 8)	Risk by 1 unit increase	97 (13.1%)	739	1.64 (1.21–2.21)	0.0014	0.0016 Neg	0.63	1.37 (1.13–1.65)	3

PH = proportional hazard; SD = standard deviation

Table 2 also presents standardized HR, expressed per 1 SD increase or decrease, and the ranking of different predictors for incidence of IHD based on these standardized HRs.

### Risk factor model

Model A including only the Framingham risk score showed a HR of 1.36 (95% CI 1.24–1.50, *p* < 0.0001) for predicting IHD. Based on the stepwise regression the significantly independent predictors among known risk factors that increase the risk of IHD were lower HDL cholesterol, higher total cholesterol and hypertension (model B). Model C included additionally categorized hs-TNT (≤ 3, 3–10, > 10 ng/L) as significant predictor. Models A, B and C are presented in Table 3.

**Table 3:** Cox proportional hazards models, A (univariable), B and C (multivariable) for time to first of MI/Unstable angina/PCI/CABG during 21 years of follow-up

Model	N	Predictors		Hazard Ratio		
				(95% CI)	*p* value	AUC (95% CI)
A	707	Framingham score	Risk by 1 unit increase	1.36 (1.24–1.50)	< 0.0001	0.69 (0.63–0.74)
B	710	HDL cholesterol (mmol/L)	Risk by 0.2 units decrease	1.48 (1.26–1.73)	< 0.0001	0.71 (0.66–0.77)
		Hypertension	Yes vs No	2.14 (1.41–3.24)	0.0003	
		Total cholesterol (mmol/L)	Risk by 1 unit increase	1.43 (1.17–1.74)	0.0004	
C	710	HDL cholesterol (mmol/L)	Risk by 0.2 units decrease	1.49 (1.27–1.75)	< 0.0001	0.72 (0.67–0.78)
		Hypertension	Yes vs No	2.05 (1.35–3.10)	0.0008	
		Total cholesterol (mmol/L)	Risk by 1 unit increase	1.43 (1.17–1.74)	0.0005	
		High-sensitivity Troponin-T ≤ 3/3–10/ > 10 (ng/L)	> 3–10 vs ≤ 3	1.49 (0.90–2.45)	0.12	
			> 10 vs ≤ 3	2.96 (1.35–6.49)	0.0066	

### Predictive and discriminative ability, internal validation and calibration

Model C provided significantly better prediction than model B, as tested by likelihood ratio test (*p* = 0.033).

**Fig. 1:**
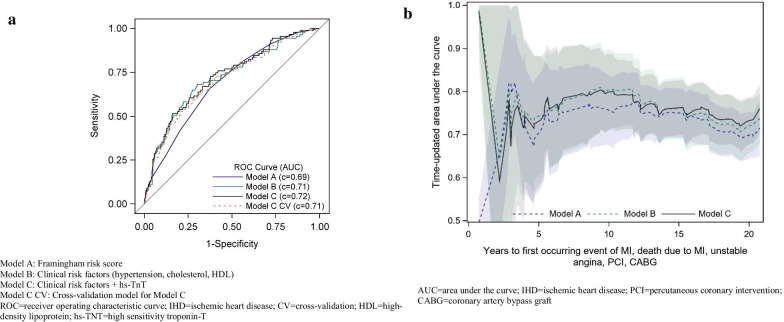
**a** ROC curve describing the predictive ability for models A (univariable), B and C (multivariable) for IHD. Model A: Framingham risk score. Model B: Clinical risk factors (hypertension, cholesterol, HDL). Model C: Clinical risk factors + hs-TnT. Model C CV: Cross-validation model for Model C. ROC = receiver operating characteristic curve; IHD = ischemic heart disease; CV = cross-validation; HDL = high-density lipoprotein; hs-TNT = high sensitivity troponin-T. **b** Time-dependent AUCs describing the predictive ability for models A (univariable), B and C (multivariable) for IHD continuously over follow-up time. AUC = area under the curve; IHD = ischemic heart disease; PCI = percutaneous coronary intervention; CABG = coronary artery bypass graft

The discriminative ability of prediction beyond 20 years of the three models was evaluated by the AUC that was 0.69 (95% CI 0.63–0.74) for model A, 0.71 (95% CI 0.66–0.77) for model B, and 0.72 (95% CI 0.67–0.78) for model C by further adding the categorized hs-TNT variable, Fig. 1a. No statistically significant difference was found between the three ROC curves. Time-dependent AUCs for the three models are shown in Fig. 1b.

An internal validation of model C, evaluated by cross-validation had similar AUC as observed for the main model, 0.71 (95% CI 0.65–0.76).

**Fig. 2:**
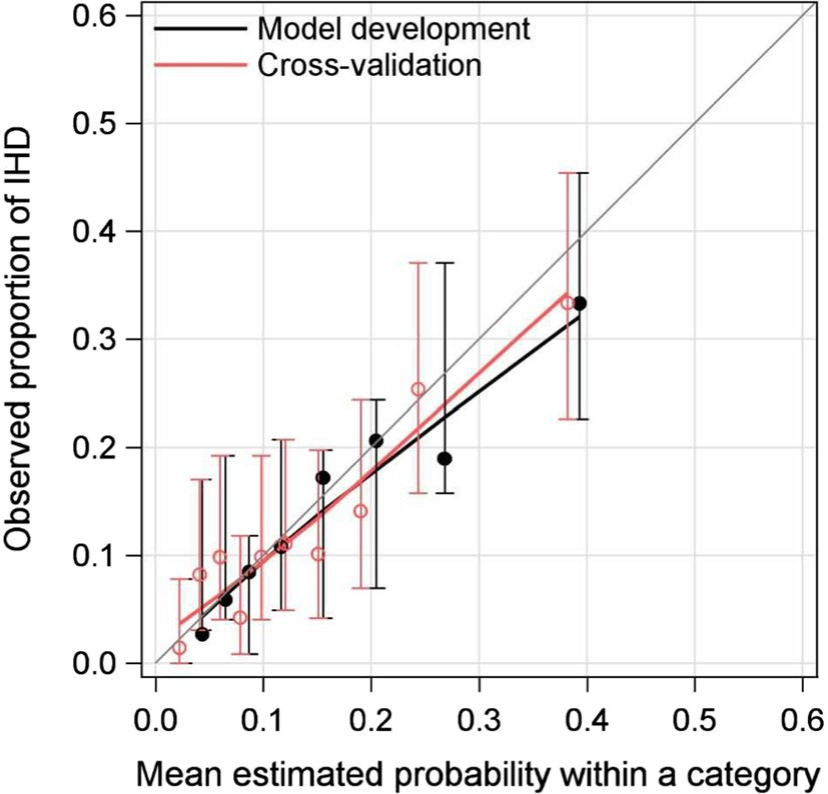
Calibration plot for model C and cross-validation for model C

Based on the estimated probabilities, the cut-offs optimising sensitivity and specificity were calculated. For model A, the sensitivity was 65.9% and the specificity 62.5%, for model B, 68.1% and 69.8%, and for model C, 72.5% and 62.4% respectively. The main and cross-validated model C was well calibrated in overall terms, as shown in Fig. 2 by the observed vs the estimated probabilities.

## Discussion

In this random population of middle-aged men without prior IHD followed for 21 years we found that, when biomarkers were studied separately, the number of high levels of biomarkers (hs-TNT, hs-CRP, IL-6 and NT-proBNP) is a significant predictor that provided highest predictive ability. In a multivariable prediction model**,** the addition of biomarker hs-TNT was an independent significant predictor of IHD and significantly improved prediction as tested by likelihood ratio test (*p* = 0.033). Furthermore, despite that addition of biomarker hs-TNT on top of clinical risk factors did not significantly enhance discriminative ability for IHD, it appeared to promote early prediction of incident IHD during 21-year long follow-up.

Previous risk scores are mostly based on clinical risk factors. The Framingham Risk Score (FRS) is a well-known, sex-specific multivariable risk factor algorithm [15]. This risk score was based on only 12 years of follow-up and only traditional risk factors, including age, total cholesterol, HDL, systolic BP, smoking and diabetes, but not including physical activity, BMI and biomarkers. Similarly, the Systematic Coronary Risk Evaluation (SCORE) is based on traditional risk factors and estimates only the 10-year risk of fatal cardiovascular diseases [16]. The QRISK (risk score using the QRESEQRCH database) incorporates family history and social deprivation and calibrates better to the UK population but their clinical CVD events were not adjudicated [17]. It can be argued that the above-mentioned risk prediction models might provide only a limited incremental risk of IHD in the general population in today´s clinical practice as they may not fully reflect the current observations of markedly decreasing population levels of smoking, and serum cholesterol, but instead an increase in overweight and obesity [18]. In our study we found that for risk estimation 20 years beyond FRS alone is a suboptimal clinical prediction model for IHD with an AUC about 0.69 in a population with fairly low cardiovascular risk profile. In the composition of FRS, HDL-C appears to be one of the most important factors, at least when FRS is applied in 50-years old men and their first event of IHD studied for approximately 20 years. Addition of further new biomarkers such as hs-TNT on top of clinical factors did improve the prediction, but did not significantly enhance discriminative ability. Together they are able to achieve AUC 0.72 which is more acceptable, as an acceptable c-statistics of at least 0.70 is recommended in clinical praxis. Furthermore, they appear to contribute to higher sensitivity for the similar levels of specificity throughout the follow-up, indicating a better prediction of IHD than FRS alone.

Atherosclerosis is a multifactorial process that involves many underlying components such as increased serum LDL-cholesterol, lipid oxidation and inflammation [1], and develops with preceding subclinical events. It is therefore reasonable to emulate this complex and multifactorial nature of the atherosclerotic process by combining different biomarkers representing different mechanisms. Indeed, when biomarkers were studied alone, the number of elevated levels of biomarkers (hs-TNT > 10 ng/L, hs-CRP > 1 mg/L, IL-6 > 8 ng/L and NT-proBNP > 100 pg/mL) is a significant predictor that provides highest predictive ability. Additionally, hs-TNT, hs-CRP and IL-6 were significant predictors for incidence of IHD. Hs-TNT has better prediction than smoking, BMI and glucose, and is an independent significant predictor when adjusted for HDL-C, cholesterol and hypertension. Also, the risk increases continuously with increasing units of the biomarker TnT indicating that this biomarker has a linear association with increased risk of IHD. Furthermore, HDL-C that is also a biomarker and widely used as a traditional risk factor, has the highest predictive ability compared with other known risk factors and the biomarkers investigated within this study. It is notable that this study involves a very long follow-up of 21 years which permit exploring long-term prediction of different biomarkers analyzed in a very early stage of disease process. A combination of different biomarkers made it possible to represent different mechanisms involved in the disease progression. It might be so that different underlying mechanisms are involved at different stages of the atherosclerotic process, and as a result the effect of biomarkers can differ along the way.

Previous studies have also studied similar biomarkers and often focused on a single biomarker for its prediction of the incidence of IHD. For instance, De Lemos et al. demonstrated that 25% of adults in the general population had detectable TnT levels (≥ 0.003 ng/mL) and that this was associated with structural heart disease and the risk of all-cause mortality during 6.4 years of follow-up [19], findings that were consistent in a subsequent meta-analysis [20]. Cesari et al. studied three inflammatory biomarkers, CRP, IL-6 and tumour necrosis factor-α, in 2225 individuals without previous cardiovascular disease. All the inflammatory biomarkers predicted cardiovascular events and the risk was highest among participants where all three inflammatory biomarkers were measured in the highest tertile [21]. In addition, other studies and meta-analysis show associations between inflammatory markers and ischaemic heart disease [2, 22, 23], but these findings have not been consistent [24] and the association can depend on conventional risk factors and additional inflammatory markers [25]. Furthermore, Daniels et al. studied the prediction of all-cause mortality and cardiovascular mortality by TNT and NT-proBNP in a cohort of 957 older adults and community residents in southern California. This study showed that participants with detectable TNT (≥ 0.01 ng/ml) and NT-proBNP (≥ 450 pg/ml) ran a higher risk of death [9]. However, to the best of our knowledge, whether these biomarkers are sensitive enough for the early prediction of IHD events at the age of 50 years in the general population has not been adequately studied previously.

Our study extends previous findings in two respects; firstly, we sought to determine the predictive value for incident IHD of combining different biomarkers representing different mechanisms such as inflammation (hs-CRP, ferritine, IL-6), cardiac stress (NTpro BNP) and cardiac damage (hs-TnT). In this study, a combination of different biomarkers provided the most powerful prediction of IHD in a univariable analysis. Secondly, we also sought to establish a risk factor model by adding biomarkers to clinical risk factors (hypertension, smoking, cholesterol*,* diabetes) and contemporary cardiovascular risk factors (BMI, physical inactivity). This strategy has previously been studied but without achieving consistent results. Zethelius et al. studied the prediction of cardiovascular death in elderly men (71 years) by a combination of biomarkers in addition to established risk factors and found that risk stratification was improved [12]. Wang et al. evaluated men and women (mean age 59 years) with a follow-up time of up to 10 years and found that a multimarker score only moderately increased the predictive value of major cardiovascular events when added to conventional risk factors [11]. Although not fully comparable, Kim et al. found similar results in a case–control study comprising only women [10]. In the present study, the addition of biomarker hs-TNT was an independent significant predictor of IHD and significantly improved prediction as tested by likelihood ratio test (*p* = 0.033). Furthermore, addition of biomarker hs-TNT on top of clinical risk factors appears to promote the early prediction of incident IHD during 21-year long follow-up. As compared with FRS, the AUC increased from 0.69 to 0.72 when adding biomarkers. This implies that prediction model consisting of FRS may be improved in a contemporary era of cardiovascular prevention with gradually declining incidence of IHD. Further studies with larger sample sizes are warranted. The reason why some biomarkers did not enhance further predictive power in addition to clinical risk factors in multivariable models, despite that they were significant in univarable analysis, might be lack of power in the present study. One other possible explanation is the fact that biomarkers such as hs-CRP represent mechanisms for inflammation, which might be overlapping with traditional risk factors such as HDL-C and hypertension in which inflammation is involved.

### Limitations

This study has some limitations. First, the sample size is limited and only men were included, making this cohort less generally representative. Secondly, the sample was representative of the population in Sweden of mostly Caucasian origin and we are therefore unable by default to extrapolate our results to other ethnic groups. Thirdly, all participants were at the age of 50 years old, subsequently making the comparison with the Framingham Risk Score possible only for this particular age group and gender. Furthermore, only 55% of participants invited accepted participation, with some bias towards a healthier subgroup. Lastly, the definition of diabetes was determined as blood glucose > 7.0 mmol/L, which corresponds to a value of 7.7 mmol/L in plasma glucose, which is the current measurement method. This might have led to an underestimation of participants with diabetes. However, in our definition, only one measurement was taken into consideration, while the diagnosis of diabetes usually requires two increased values.

## Conclusions

In this random, middle-aged male population sample, the addition of a biomarker hs-TNT was an independent significant predictor of IHD and adds to the early prediction of incident IHD in a multivariable model, in a general male population with lower risk than in previous generations. Further studies with long-term risk estimation are warranted giving consideration that the incidence rate of IHD is continuously declining.

## Data Availability

The dataset supporting the conclusions of this article are included within the article. The datasets during and/or analyzed during the current study available from the corresponding author on reasonable request.

## Abbreviations

BMI: Body mass index
CABG: Coronary artery bypass graft
hs-CRP: High-sensitivity C-reactive protein
hs-TNT: High-sensitivity Troponin T
IL-6: Interleukin-6
IHD: Ischaemic heart disease
MI: Myocardial infarction
NT-proBNP: N-terminal prohormone of brain natriuretic peptide
PCI: Percutaneous coronary intervention
