# Correction to: Multi‑modality biomarkers in the early prediction of ischaemic heart disease in middle‑aged men during a 21‑year follow‑up

**DOI:** 10.1186/s12872-021-01921-x

**Published:** 2021-02-23

**Authors:** Maria Sakalaki, Per-Olof Hansson, Annika Rosengren, Erik Thunström, Aldina Pivodic, Michael Fu

**Affiliations:** 1 Department of Molecular and Clinical Medicine, Institute of Medicine, Sahlgrenska Academy, Sahlgrenska University Hospital/Östra Hospital, University of Gothenburg, Diagnosvägen 11, 41650 Gothenburg, Sweden; 2 Department of Medicine, Geriatrics and Emergency Medicine, Sahlgrenska University Hospital/Östra, Gothenburg, Sweden; 3 Statistiska Konsultgruppen, Gothenburg, Sweden; 4 Department of Ophthalmology, Institute of Neuroscience and Physiology, Sahlgrenska Academy, University of Gothenburg, Gothenburg, Sweden

## Correction to: BMC Cardiovasc Disord https://doi.org/10.1186/s12872-021-01886-x

Following publication of the original article [1], an error was identified in in Fig. 2. The correct figure is given below.

**Fig. 2:**
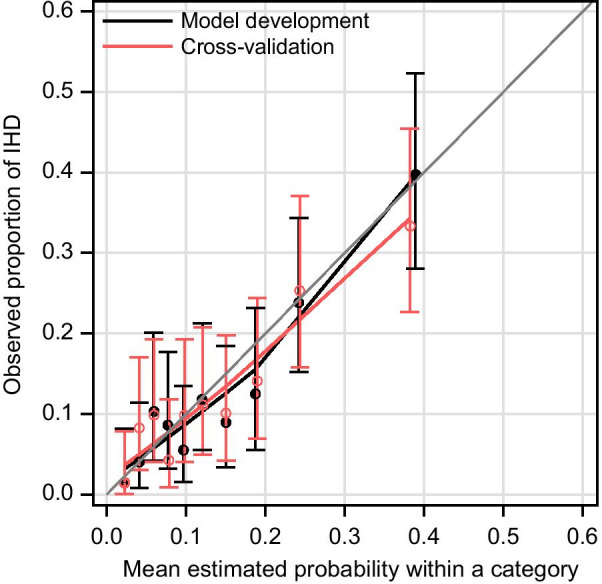
Calibration plot for model C and cross-validation for model C
